# Outcomes of Copperhead Snake Envenomation Managed in a Clinical Decision Unit

**DOI:** 10.5811/westjem.20369

**Published:** 2025-07-09

**Authors:** Mary A. Wittler, Brian Hiestand, Amlak Bantikassegn, David M. Cline, Jennifer L. Hannum

**Affiliations:** * Wake Forest University School of Medicine, Department of Emergency Medicine, Winston-Salem, North Carolina; † Atrium Health CMC, Department of Internal Medicine, Charlotte, North Carolina

## Abstract

**OBJECTIVES:**

Copperhead envenomations are the most common snakebite in the United States, and the majority are categorized as mild-moderate severity. The need for prolonged observation to monitor for signs of envenomation supports observation in a clinical decision unit (CDU). To our knowledge, no articles to date have reported on the clinical outcomes of patients managed in a snakebite CDU protocol.

**METHODS:**

We performed a five-year structured, retrospective cohort study of adult patients managed in a single-center CDU, compared to a 10-year period of historical cohort managed inpatient at the same institution. Several clinical parameters were abstracted for comparison. The primary outcome was effective management in CDU observation as measured by length of stay (LOS), disposition, and documented return for care within the hospital system. Secondary outcomes were management comparisons between groups, as measured by LOS, frequency of antivenom use and vials administered, and surgical interventions.

**RESULTS:**

The two cohorts included 59 patients on CDU observation protocol compared to 36 patients as historical inpatient management. Fifty-four patients (92%) were discharged from observation. Five patients converted to inpatient admission, mostly secondary to uncontrolled pain. After discharge, six patients in the CDU cohort (10.2%) returned for care within the network for wound checks and/or concern for extremity swelling; all were discharged. Compared to the inpatient cohort, patients managed in CDU observation had shorter LOS, less antivenom administered, and fewer surgical interventions.

**CONCLUSION:**

Copperhead snakebites can be managed effectively in clinical decision unit observation. The majority of patients were discharged from observation with few return visits. Few patients required admission; those who did were secondary to pain control issues. Anticipated gains of CDU observation are shortened length of stay and lower resource utilization.

## INTRODUCTION

In the United States, approximately 4,700 native venomous snakebites were reported to poison centers in 2020, with very few deaths.^1^ The majority of envenomations belong to the Crotalinae (crotalid) subfamily of Viperidae that includes rattlesnakes, cottonmouths, and copperheads. Of these, copperheads (genus *Agkistrodon*) are the most reported envenomation, and the majority of these envenomations (approximately 90%) are coded as minor or moderate outcomes, with only approximately 2% meeting the criteria for severe outcomes.^1^

At our institution in North Carolina, most patient presentations for snakebites are from the eastern copperhead (*Agkistrodon contortrix*). Compared to other pit vipers, copperhead envenomations are less severe, rarely causing hemodynamic compromise or clinically significant coagulopathy. The most common clinical finding is local tissue effect including swelling, ecchymosis, erythema, and/or pain.^2^–^4^ Patients may present with minor tissue effects and subsequently develop significant signs of envenomation after a latent period. Additionally, for all snakebites, approximately 25% of envenomations are dry bites, such that no venom effects are seen.^5^ Expert consensus recommends an observation period of at least eight hours to delineate dry bites, and 12–24 hours for minor envenomations.^6^

As most copperhead envenomations present with minor or no systemic symptoms and non-clinically significant hematologic effects, management is confined to treatment with antivenom based on tissue injury, pain control, and patient education. Based on expert consensus recommendations, even apparently dry or minor envenomations need prolonged observation. The need for prolonged observation and hemodynamic stability of this patient population makes it amenable to management in a clinical decision unit (CDU) snakebite-observation protocol. To our knowledge, no articles to date have reported on the utility and clinical outcomes of patients managed in a snakebite CDU protocol. Our objective was to provide descriptive data for clinical outcomes and management of copperhead bites in a CDU protocol and compare it to inpatient care outcomes. We performed a structured, retrospective chart review of patients managed in our CDU, which we then compared to an inpatient historical cohort.

## METHODS

The study design was a five-year structured, retrospective cohort chart review^7^ of adult patients managed in a single-center CDU snakebite protocol (2013–2017), compared to a 10-year period of a historical cohort managed as inpatients at the same institution (2003–2012). We identified medical records as described below by querying the hospital electronic health records (EHR) database (Epic Systems Corporation, Verona, WI). This study was approved by the institutional review board.

Adult snakebite patients were considered CDU candidates based on copperhead identification by the patient, family member, or clinician and hemodynamic stability. We excluded patients with active comorbid illnesses CDU observation status based on predetermined criteria. Specific monitoring and treatment components of the protocol were designed based on review of published snakebite observational protocols and management algorithms, and expert consultation with two board-certified medical toxicologists (MW, JH). A specific observation order set was created for this population, and all advanced practice practitioners (APP) staffing the CDU were provided an educational module that reviewed treatment goals and monitoring parameters, indications for antivenom administration, goals of discharge, and follow-up care recommendations that included return precautions. The CDU clinicians could consult with local medical toxicologists and/or the regional poison center as desired.

**Population Health Research Capsule**

What do we already know about this issue?

*Copperhead envenomations need a prolonged period of observation prior to disposition*.

What was the research question?

*We performed a retrospective chart review of copperhead envenomations to demonstrate effective management in a clinical decision unit (CDU)*.

What was the major finding of the study?

*Copperhead bites managed in a CDU had a 92% discharge rate, most occurring < 24 hours with low return rates, and a 50% shorter stay compared to inpatient care*.

How does this improve population health?

*CDU observation for copperhead envenomation, the most commonly reported snakebite in the US, could result in shortened length of stay and decreased use of inpatient resources*.

For the CDU cohort retrospective chart review, the EHR was queried for patients managed with the ED/CDU snakebite envenomation observation order set. We abstracted the age and sex of patient, snake species identified, transfer status from a regional hospital, time from envenomation to initial presentation, location of injury, worst documented extremity swelling, systemic symptoms, laboratory values, antivenom administration, surgical intervention, total hours admitted, and final disposition (admitted to the hospital, discharged, left against medical advice). Abstracted systemic symptoms included the following: low blood pressure; chest pain; shortness of breath; nausea; vomiting; diarrhea; headache; diaphoresis; weakness; dizziness; and/or paresthesia. For all patients, an overall severity grading was assigned as per Table 1, based on the worst assessment of documented swelling, and consideration of hematologic and systemic symptoms. This grading scale is a minor modification of standard criteria, such that entire extremity swelling is categorized as severe.^8^

We use Crotalidae Polyvalent Immune Fab Ovine (FabAV) (BTG International Inc, West Conshohocken, PA) do not routinely administer maintenance FabAV vials for copperhead envenomation. Thus, for FabAV dosing, we abstracted total number of vials administered to gain control of envenomation (defined as cessation of continued tissue swelling), and any further vials administered. We recorded worst hematologic laboratory abnormalities. Acute hypersensitivity reactions (rash, urticaria, dyspnea, angioedema, hypotension) temporally related to administration of FabAV were documented. Additionally, we reviewed whether any patients discharged presented again at any of our network hospitals for concerns related to the envenomation and ultimate disposition. We excluded any patient who entered observation care and subsequently left against medical advice (AMA).

To determine patterns of care prior to use of CDU observation, we reviewed the records of adult patients admitted to the hospital over a 10-year period immediately preceding implementation of the snakebite observation protocol whose discharge diagnosis included “toxic effect of snake venom.” This time frame was chosen as a convenience sample based on access to the EHR for data abstraction (Older patient encounters were not reliably identifiable from the predating EHR system.) Patients were identified by *International Classification of Disease*s Rev, 9 and 10 ICD-9 and ICD-10 codes, and patient data were abstracted for similar findings. We excluded any records with snake species identified as non-copperhead, left AMA during admission, or secondary evaluation for snakebite (not primary admission). Patients were admitted to a variety of services (ie, hospitalist, family medicine, or surgical specialty). Specialty consultation with local medical toxicologists and/or the regional poison center was available if requested by the inpatient clinician.

One author (AB), who was trained in the CDU protocol, management, and snakebite severity assessment abstracted the variables into a structured Excel spreadsheet (Microsoft Corporation, Redmond, WA). One of the authors who is a medical toxicologist reviewed an initial pilot of 15 charts to confirm accuracy of data abstraction. He periodically met in person with the abstractor to confirm training on assignment of envenomation severity grade, and ad hoc to resolve any questions in data abstraction. Any confounders or questions related to outcome measures were resolved by a second author, who is also a medical toxicologist. We included any case with missing variables in the analysis, and missing variables were noted in the statistical analysis.

The primary outcome of the CDU protocol was effective management of this patient population in CDU observation status. Typical ED CDU outcome metrics were chosen,^8^ including length of stay (LOS), final disposition (discharge or inpatient conversion), and ED return for recheck after discharge (with final disposition) at the main or affiliate hospitals. Secondary outcome measures were comparisons between cohorts for the number of patients treated with antivenom, number of FabAV vials administered, LOS, surgical interventions, and return for recheck (with final disposition).

We analyzed the data using SAS 9.4 (SAS Institute Inc, Cary, NC). Chi-square test or the Fisher exact test were used as appropriate to compare frequencies of categorical variables between the CDU and historical cohorts. Medians and Kruskal-Wallis tests were used to compare continuous variables between groups. Formal power analysis was not performed, as the cohort size was fixed by the time intervals examined. An alpha of 0.05 was held to be statistically significant.

## RESULTS

**Figure 1:**
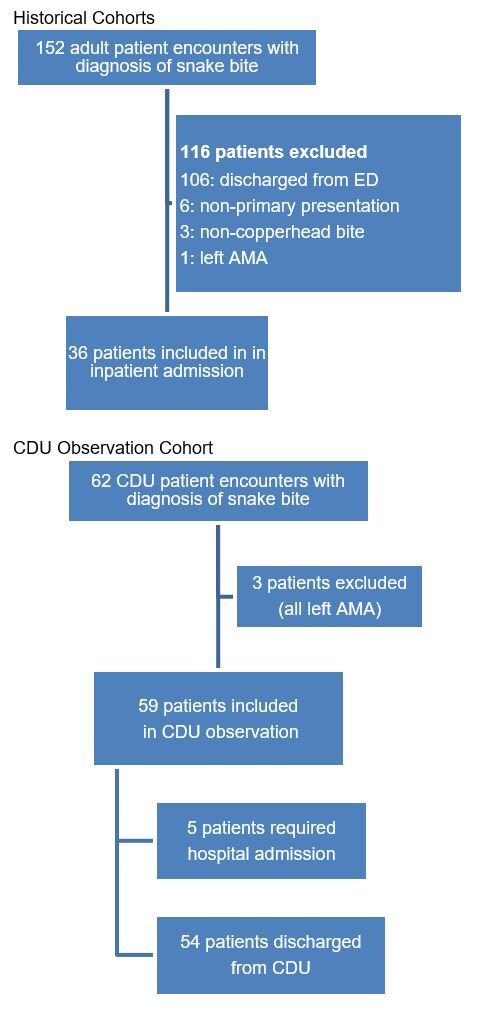
Patient encounters screened in a study of copperhead envenomations managed in a clinical decision unit vs. inpatient care.

For the two cohorts, we screened all patient encounters for inclusion and exclusion criteria, resulting in encounters that were further chart reviewed, as shown Figure 1.

The two groups included 59 patients in the CDU cohort vs 36 patients in the historical cohort. Baseline demographics for patients and bite characteristics are shown in Table 2.

All evaluated parameters were balanced between groups, with no significant statistical difference between groups. While there were slightly more males and upper extremity bites in the historical cohort compared to the CDU cohort, these were not statistically significant. For the parameters of age and time since envenomation to presentation, the results were not a normal distribution; nonparametric analysis showed no significant difference.

Systemic symptoms and hematologic abnormalities are shown in Table 3.

For the primary outcome of effective CDU observation care, we reviewed observation LOS, disposition, and ED revisitation rates. The median LOS in observation care was 16 hours (see Table 4). For disposition, 92% of patients were discharged from observation. Five patients of moderate-severe clinical severity were converted to inpatient management: four patients secondary to continued pain (one patient with a severe lower extremity bite, three patients with moderate severity hand bites), and one patient secondary to temporary closure of the CDU. No patient received further antivenom administration as an inpatient. Of patients discharged home from the CDU, 10.2% returned to one of our network emergency departments (ED) for wound check and/or concern for extremity swelling. All exams were uncomplicated and reassuring, and all patients were discharged from the ED. We had one minor deviation from protocol conditions in which a nonstandard dosing of antivenom (three vials) was administered as no more was available at the affiliate hospital at time of the infusion. Per protocol, no patient automatically received maintenance dosing of antivenom. One patient received a subsequent dose of two vials of antivenom for treatment of recurrent envenomation symptoms.

When comparing secondary outcome measures between the cohorts, it became apparent that the inpatient historical cohort had a higher percentage of moderate and severe envenomations. The overall distribution of clinical severity categorized as mild, moderate, or severe between the CDU cohort vs the historical cohort were 30.5%, 52.5%, and 17.0% vs 8%, 75%, and 17%, respectively. Because of this uneven distribution, all comparisons between cohorts incorporated clinical severity.

Initial comparison between cohorts included the frequency of FabAV administration and number of vials administered. The total number of patients that received FabAV, stratified by severity of envenomation and treatment group, is shown as frequency of FabAV administration in Table 5.

Overall, FabAV was administered less frequently in the CDU cohort compared to historical cohort (45.76% vs 77.78%). To account for the unequal clinical severity distribution between cohorts, a secondary analysis (not represented) that excluded mild envenomations from the CDU cohort found no significant difference in the number of patients treated with FabAV between cohorts (63.41% of CDU cohort vs 75.76% historical cohort, *P* = .25). Further analysis based on clinical severity showed that compared to historical cohort, patients cared for in the CDU cohort were significantly less frequently treated with antivenom in the mild and moderate groups (Fishers exact test, *P* < .01 and *P* = 0.02, respectively), and more frequently treated with antivenom in the severe group (Fisher exact test, *P* = 0.04).

Additional comparisons between cohorts included LOS, surgical interventions, and return for recheck. Length of stay for observation in the CDU vs inpatient LOS is represented in Table 4. When comparing LOS between the CDU cohort vs the historical cohort, the median LOS in hours (hrs) was statistically significantly shorter (16 vs 32 hrs, respectively, Kruskal-Wallis, *P* < .001). Even after mild envenomations were excluded from analysis, the LOS remained significantly shorter for the CDU cohort compared to the historical cohort (Kruskal-Wallis, *P* < .001). When reviewing LOS stratified by envenomation severity, the CDU cohort LOS was significantly reduced for mild and moderate envenomation compared to historical cohort (see Table 4). While severe envenomation LOS in the CDU group was slightly shorter, this was not statistically significant.

Four cases of surgical interventions occurred in the historical cohort: two incision and drainage of hand bites with wound vac placement, and two decompressive fasciotomies of upper extremities. No patients in the CDU protocol group received surgical intervention. When comparing second ED visits for wound check, there was no statistically significant difference between cohorts with six (10.2%) CDU cohort patients vs two (5.56%) historical cohort patients returning to the ED. In both cohorts, all patients were discharged, and no specific interventions were provided.

## DISCUSSION

To our knowledge, no previous publication provides descriptive outcomes of snakebite patients managed in a CDU protocol or compares management outcomes to similar type of envenomation managed in inpatient care. Copperhead envenomations are the most common snakebites at our institution and are usually devoid of clinically significant bleeding or systemic effects. Expert consensus recommends a prolonged period of observation of snakebites prior to disposition. For copperhead bites, the goals of care focus on monitoring of tissue effects, treatment of pain, and administration of antivenom when indicated. Thus, copperhead bites are good candidates for CDU observation.

Based on a five-year review of CDU observation data, copperhead bites can be effectively managed in a CDU protocol. The standard time for ED CDU observation care is <24 hours, and most studies show that the approximate LOS for observation care is approximately 15 hours. A general guideline for observation care is a discharge rate of 80% with inpatient conversation of 20%.^9^ In our CDU cohort, the majority (92%) of patients were discharged from observation care with a median LOS of 16 hrs, and all patients deemed stable for discharge were dispositioned < 24 hrs. Of the five patients converted to inpatient admission, the indication for admission was for continued pain management or unanticipated closure of the CDU. In review of the charts of patients admitted for pain, onr patient had a severe lower extremity bite, and three had moderate severity hand bites. None required further antivenom or specific interventions. After discharge, few patients (10.2%) bounced back within the network hospital system for re-evaluation of their snakebite wound or extremity swelling. All patients had reassuring exams and were discharged from the ED without intervention. We did not have any readmissions within our network hospitals after discharge. We had minimal deviation from the condition-specific treatment protocol.

Comparing the CDU cohort to the historical inpatient cohort, the most clinically impactful finding was that patients managed in CDU observation had a statistically significantly lower LOS. For all patients, the CDU observation cohort vs historical inpatient cohort LOS was 16 hrs vs 32 hrs, respectively. Even after adjusting for the larger proportion of mild envenomations in the CDU observation cohort, the LOS remained significantly shorter for the CDU cohort compared to the historical cohort. Despite this decreased LOS, there was no statistically significant difference between cohorts for patients returning to the ED for wound check or wound complications. While we found other interesting statistically significant findings, such that CDU patients were significantly less likely to receive antivenom for mild-to-moderate envenomation, and more likely to receive antivenom for severe envenomations, the clinical significance is unknown. This could be related to a more unified care practice delivered in the CDU secondary to protocolized care and APP training. Of note, both cohorts had availability to specialized consultation with medical toxicologists and/or local poison center, under the discretion of the cinician directing patient care.

Our CDU observation patient population aligns with published snakebite victim demographics. As in other studies, most snakebite patients were male, with predominantly upper extremity bites.^2^,^10^ As previously published, gastrointestinal symptoms were the most common systemic effect after copperhead bites,^2^ although we did not track whether symptoms occurred in relation to opioid administration. We found low rates of minor coagulopathy, consistent with published literature for copperhead bites.^2^ Based on our modified severity grading, 30.5%, 52.5%, and 17% were classified as mild, moderate, and severe, respectively. Direct comparison to other published copperhead envenomation is difficult secondary to non-uniform scoring of extremity swelling and grading of overall clinical severity. In one retrospective poison center study, 33% of admitted copperhead bites developed swelling of greater than half of the envenomated extremity.^11^ A second retrospective study of southern copperhead bites found that 85% of patients developed only local swelling.^10^ By comparison, our patients trended toward a more severe envenomation based on local tissue injury.

General recommendations for FabAV administration include progressive signs or symptoms after a crotaline snakebite.^6^ The package insert recommends initial dosing of 4–6 vials, to be repeated until control has been achieved, followed by maintenance therapy (2 vials every 6 hours for 3 doses).^12^ Maintenance vials are not routinely administered at our institution for copperhead envenomations, and the use of empiric maintenance therapy varies across institutions.^6^ Forty-six percent of our protocol patients were treated with FabAV; the median dose of antivenom to obtain control was four vials [range 3–14 vials]. In comparison, a 2007 poison center study showed that approximately 36% of copperhead bites were treated with antivenom, with the trend of increasing administration over the study period.^13^ A 10-year review of copperhead snakebites reported to Ohio poison control centers through 2016 showed that 45% of patients were treated with antivenom and the frequency of antivenom use did not increase over time.^14^ In contrast to poison control center data, a recent hospital-based retrospective review looking at predictors of FabAV use in copperhead envenomation found that 75% of patients received antivenom with a median number of 10 vials given.^15^ For copperhead envenomations, total FabAV to obtain control typically averages 4–6 vials (range 4–10).^2^,^4^,^16^ An incidental finding was the low rate of reaction to antivenom, similar to recent published rates for FabAV.^17^,^18^

Using ED CDU observation care for snakebite management resulted in significantly lower LOS compared to LOS reported in the literature. In one retrospective study of admitted copperhead envenomations, patients with swelling of less than half of the envenomated extremity or greater than or equal to half of the envenomated extremity, LOS was 1.7 days (range 1–5) or 3.5 days (range 1–7), respectively.^11^ In a separate study of admitted copperhead envenomations, average LOS was 40 hrs.^4^ In the North American Snakebite Registry, approximately 44% of copperhead bites were admitted for less than 24 hrs.^2^ Our findings support utilization of CDU observation for copperhead envenomations to minimize inpatient resource utilization and decrease LOS.

An additional potential benefit of CDU observation care is that APPs develop expertise in managing snakebites. Our APPs’ comfort level and clinical judgment has increased though years of care delivery for this diagnosis. In the absence of a CDU observation, patients may be admitted to a wide range of care units under a variety of services. As a result, clinicians are often uncomfortable taking care of patients with snakebite envenomations given the low frequency of bites overall.

## LIMITATIONS

This study has all the limitations of a retrospective chart review and includes only adult patients. During construction of the review, the abstractor was not blinded to case assignments. While the abstractor met with the author to resolve questions, we did not sample charts for expert interobserver reliability. Patients and/or family members identified the snake type, and in some instances the snake type was unknown. However, based on epidemiologic data for our area, copperhead envenomation was most likely. While there may be geographic variation of venom within the species,^19^ our results are likely generalizable to copperhead bites in other regions of the US. Despite a protocol-driven guideline for treatment, there is clinician variance in administration of FabAV, particularly in moderate envenomations.

Additionally, CDU observation excluded patients with active comorbid illness (eg, uncontrolled diabetes), and this may have impacted our comparison results, specifically LOS. There are several limitations in the historical cohort comparison. We did not track comorbid illness and/or chronic medical conditions that might impact outcome comparators, especially LOS. Additional limitations of interpreting the comparison to historical inpatient cohort include that FabAV administration was not protocolized, that clinical observations were likely under-reported, and there were very few mild envenomations. We suspect that many of the envenomations were observed in the ED, classified as mild, and subsequently discharged; thus, this population would not have been captured in our study design and possibly introduced bias into the results. We attempted to account for the heterogeneity of the data in our statistical analysis by taking into account the worst recorded severity of envenomation during comparisons.

For both cohorts, consultation with local poison control and/or medical toxicology was at the discretion of the admitting clinician. Thus, although specialty resources were available for both cohorts, differences in LOS and/or frequency of FabAV administration may have been impacted by resource utilization for the CDU cohort, including protocolized care, education of APPs providing observation care, and/or medical toxicology consultation. Therefore, our CDU outcomes might not be applicable to all institutions. While our CDU dataset does not extend beyond 2017, we do not believe that this significantly impacted our findings. We have not made changes to our CDU protocol since onset, our regional indication for FabAV administration and/or dosing has not changed, and expert guidance for snakebite management has not significantly altered.

## CONCLUSION

Based on our review, copperhead snakebites can be effectively managed in ED CDU observation care using outcome measures of LOS and low rate of conversion to inpatient admission. In our observation unit, we discharged the majority of patients from observation care within 24 hours. Patients who were admitted after observation were admitted for pain control only. We had few patients return for care within our network hospital system after discharge for re-evaluations of their wounds; all were discharged from the ED after reassuring examinations without specific interventions. Anticipated gains of ED CDU observation care for copperhead bites include shortened length of stay and decreased utilization of inpatient resources.

## Figures and Tables

**Table 1:** Snakebite severity grading scale in a study of copperhead envenomations managed in a clinical decision unit vs. inpatient care.

Mild	Local swelling, pain, ecchymosis
Moderate	Swelling less than full extremity
	Non-life-threatening systemic signs (nausea, vomiting, etc.)
	Non-significant coagulation study abnormalities
Severe	Swelling of the entire extremity
	Systemic illness (AMS, severe hypotension, respiratory insufficiency, serious bleeding or significant coagulation abnormality)

*AMS*, altered mental status.

*ED*, emergency department; *CDU*, clinical decision unit; *AMA*, against medical advice.

**Table 2:** Patient demographics and characteristics in a study of copperhead envenomations managed in a clinical decision unit vs. inpatient care.

Characteristics	CDU Cohort	Historical Cohort	Statistical Difference P-value
N	59	36	
Male, N (%)	34 (57.63)	26 (72.22)	.15†
Age, median year (range)	41 (20 – 86)	47.5 (19 – 82)	.35‡
Time since envenomation, median hour (range)	2 (0.25 – 28)	3* (0.5 – 30)	.11‡
Transfer from OSH, %	35.59	41.67	.55†
Anatomic bite location, N (%)			.28†
Upper extremity	31 (52.54)	23 (63.89)	
Lower extremity	28 (47.46)	13 (36.11)	
Snake type identified, N (%)			.81†
Copperhead	48 (81.36)	30 (83.33)	
Unknown	11 (18.64)	6 (16.67)	

† Chi-square test,

‡ Kruskal-Wallis test.

* Time could not be determined in one patient based on chart review .

*CDU*, clinical decision unit; *OSH*, outside hospital.

**Table 3:** Systemic symptoms and hematologic abnormalities in a study of copperhead envenomations managed in a clinical decision unit vs. inpatient care.

Symptoms	CDU Cohort N (%)	Historical Cohort N (%)
Nausea	12 (19.7)	2 (5.6)
Vomiting	2 (3.3)	1 (2.8)
Chest tightness or pain	2 (3.3)	0
Dyspnea	1 (1.6)	0
Hypotension	2 (3.3)	1 (2.8)
Headache	2 (3.3)	0
Dizziness	4 (6.6)	0
Platelet count < 150/mL	7 (12.7)*	4 (7.3)**
INR >1.2	5 (9.1)*	6 (17.1)**
Rash post FabAV	0	3 (8.3)

* Data available for 55 of 59 patients,

** Data available for 35 of 36 patients.

Gastrointestinal effects were the most common systemic effect, occurring in 20% of CDU patients. A minor number of patients had other systemic symptoms. A small number of patients had abnormal prothrombin time and/or abnormal platelets. No cases of hematologic abnormalities were clinically significant.

*CDU*, clinical decision unit; *FabAV*, Crotalidae Polyvalent Immune Fab Ovine.

**Table 4:** Length of stay in a study of copperhead envenomations managed in a clinical decision unit vs. inpatient care.

Severity	CDU Cohort LOS hours, median (range)	Historical Cohort LOS hours, median (range)	Statistical Significance*
All	16 (2.3 – 105.5)	32 (13.5 – 94.5)	*P* < .001
Mild	9.9 (2.3–22)	32.5 (16 – 70)	*P* = .01
Moderate	18.5 (6.8–105.5)	36 (14 – 94.5)	*P* < .001
Severe	21.1 (11 – 85)	23.6 (13.5 – 66)	*P* = 1

* By Kruskal-Wallis test.

*LOS*, length of stay; *CDU*, clinical decision unit.

**Table 5:** Frequency of FabAV administration stratified by cohort and severity in a study of copperhead envenomations managed in a clinical decision unit vs. inpatient care.

Severity	All patients receiving FabAV/Total patients (%)	CDU patients receiving FabAV/Total patients (%)	Historical patients receiving FabAV/Total patients (%)	OR (95% CI)✝	P-value
Mild envenomation	4/21 (19.1)	1/18 (5.6)	3/3 (100.0)	0.06 (0.01–0.37)	<.01*
Moderate envenomation	40/58 (69.0)	17/31 (54.8)	23/27 (85.2)	0.21 (0.06–0.76)	.02*
Severe envenomation	11/16 (69.0)	9/10 (90.0)	2/6 (33.3)	18 (1.24–260.92)	.04*
All envenomation	55/95 (57.9)	27/59 (45.8)	28/36 (77.8)	0.24 (0.09–0.62)	< .01†

✝ OR with 95% CI reflects change since protocol implementation,

* Statistically significant difference by Fisher exact test,

† Statistically significant difference by chi-square test.

*FabAV*, Crotalidae Polyvalent Immune Fab Ovine; *CDU*, clinical decision unit; *CI*, confidence interval; *OR*, odds ratio.
